# Type II collagen antibody response is enriched in the synovial fluid of rheumatoid joints and directed to the same major epitopes as in collagen induced arthritis in primates and mice

**DOI:** 10.1186/ar4605

**Published:** 2014-07-08

**Authors:** Ingrid Lindh, Omri Snir, Erik Lönnblom, Hüseyin Uysal, Ida Andersson, Kutty Selva Nandakumar, Michel Vierboom, Bert 't Hart, Vivianne Malmström, Rikard Holmdahl

**Affiliations:** 1 Department of Medical Biochemistry and Biophysics, Section for Medical Inflammation Research, Karolinska Institutet, SE-171 77 Stockholm, Sweden; 2 Department of Medicine, Rheumatology Unit, Karolinska University Hospital, SE-171 76 Stockholm, Sweden; 3 Department of Immunobiology, Biomedical Primate Research Centre, 2280 GH Rijswijk, The Netherlands; 4 Present address; Department of Immunology, Centre for Immune Regulation, Oslo University Hospital-Rikshospitalet, University of Oslo, 0424 Oslo, Norway; 5 Present address; Department of Molecular Biology and Genetics, Çanakkale Onsekiz Mart Üniversity, 17020 Çanakkale, Turkey

## Abstract

**Introduction:**

Antibodies towards type II collagen (CII) are detected in patients with rheumatoid arthritis (RA) and in non-human primates and rodents with collagen induced arthritis (CIA). We have previously shown that antibodies specific for several CII-epitopes are pathogenic using monoclonal antibodies from arthritic mice, although the role of different anti-CII epitopes has not been investigated in detail in other species. We therefore performed an inter-species comparative study of the autoantibody response to CII in patients with RA versus monkeys and mice with CIA.

**Methods:**

Analysis of the full epitope repertoire along the disease course of CIA was performed using a library of CII triple-helical peptides. The antibody responses to the major CII epitopes were analyzed in sera and synovial fluid from RA patients, and in sera from rhesus monkeys *(Macaca mulatta)*, common marmosets *(Callithrix jacchus)* and mice.

**Results:**

Many CII epitopes including the major C1, U1, and J1 were associated with established CIA and arginine residues played an important role in the anti-CII antibody interactions. The major epitopes were also recognized in RA patients, both in sera and even more pronounced in synovial fluid: 77% of the patients had antibodies to the U1 epitope. The anti-CII immune response was not restricted to the anti-citrulline protein antibodies (ACPA) positive RA group.

**Conclusion:**

CII conformational dependent antibody responses are common in RA and are likely to originate from rheumatoid joints but did not show a correlation with ACPA response. Importantly, the fine specificity of the anti-CII response is similar with CIA in monkeys and rodents where the recognized epitopes are conserved and have a major pathogenic role. Thus, anti-CII antibodies may both contribute to, as well as be the consequence of, local joint inflammation.

## Introduction

Rheumatoid arthritis (RA) is an autoimmune, chronic inflammatory disorder affecting peripheral joints. RA is a complex disorder believed to consist of different pathogenetic mechanisms that may lead to common final pathways and shared clinical signs and symptoms. Recent progress has strengthened the view that a pathogenic process leading to RA starts many years before the clinical onset. Individuals with a genetic predisposition, mainly based on certain MHC class II alleles, develop an antibody response to citrullinated proteins (anti-citrullinated protein antibodies (ACPA)) and to IgG (rheumatoid factors) combined with a raised systemic inflammatory response. Many years later the joints are affected. During the pre-RA period the autoantibody responses, including ACPA, is increased in titers and spreads in epitope specificity [1] and that antibodies to cartilage can be measured around clinical onset [2-4], where one of the autoantibody targets is collagen type II (CII). Over the years there has been a large discrepancy in the rate of the CII-specific response, ranging from a few percent up to 50% [3,4]. Also unclear is whether the low titers can be of pathogenic or regulatory relevance. These variable results are probably due to technical artifacts due to the usage of solid-phase assays with crudely purified matrix protein.

Immunization of CII in animals gives rise to significant titers of autoantibodies to CII. Three CII-specific monoclonal antibodies (CII-C1, UL-1, and M2139) have been raised and characterized in detail by the molecular interaction with their target molecule [5-8], binding *in vivo*[9], and their pathogenic and regulatory importance [10]. It would therefore be of value to directly compare human antibody specificities with the specificity of autoantibodies in animals and also to develop assays that are not dependent on collagen prepared from tissue complexed with not very well defined matrix molecules, and instead use synthetic and recombinant triple-helical CII peptides containing identified epitopes known to be exposed and recognized by antibodies *in vivo*. The purpose of this study was thus to investigate the epitope repertoire of the major CII epitopes and to analyze these not only in RA but also in a comparative way in other species; that is, in monkeys and mice with collagen-induced arthritis (CIA).

## Materials and methods

### Rheumatoid arthritis patients and healthy subjects

Serum and synovial fluid (SF) samples were collected from a previously described patient cohort [11] with established RA (290 patients, mean age 55, age range 21 to 86, 82% female) according to the American College of Rheumatology criteria [12]. For serum analysis of the C1 epitope, an additional 124 patients were included, giving a total of 414 patients (mean age 59, age range 21 to 100, 80% female). All patients attended the Rheumatology Clinic at the Karolinska University Hospital, Sweden, and were included based on a clinical need for withdrawing SF from knee effusions. Serum sampling was performed in parallel. One hundred serum samples from healthy subjects (mean age 54, range 24 to 82, 81% female) were also analyzed and served as controls in this study. The ethical review board of the Karolinska University Hospital (Regionala Etikprövningsnämnden Stockholm) approved this study, and all subjects gave informed consent.

### Nonhuman primates and mice

The Biomedical Primate Research Centre in Rijswijk, the Netherlands supplied sera from both rhesus monkeys (*Macaca mulatta*) and common marmosets (*Callithrix jacchus*)*.* The monkeys were at adult age, rhesus monkeys >4 years and common marmosets >1.5 years, when the experiments were performed. In accordance with the Dutch law on animal experimentation, all study protocols in which these animals took part were reviewed and approved by the Institute’s Ethics Committee (Biomedical Primate Research Centre) before the experiments started (application numbers 589, 605, 613/618, 633, 484, 542, 581). The B10.Q/rhd mice originated from Professor Jan Klein, Tübingen, Germany, whereas the BALB/cJ mice originated from Jackson Laboratories (Bar Harbor, ME, USA). The parental inbred strain as well as the B10.Q × (BALB/c × B10.Q) N2 strain were bred in the animal facility of the Institute of Medical Inflammation Research at Lund University, Sweden. The animals were between 8 and 12 weeks old at the start of the experiments. Stockholm norra försöksdjursetiska kommiten, Stockholm, Sweden (permission number N66/10, N169/10) approved the experiments.

### Induction and evaluation of CIA in rhesus monkeys and common marmosets

Rhesus monkeys were immunized with chicken-derived CII (MD Biosciences, Zürich, Switzerland) and common marmoset monkeys were immunized with either chicken or bovine-derived CII (MD Biosciences), which were dissolved in 0.1 M acetic acid to a final concentration of 10 mg/ml (for rhesus monkeys) and 5 mg/ml (for common marmosets) and mixed with an equal volume of complete Freund’s adjuvant (Difco, Detroit, MI, USA). CIA was induced by injection of 1 ml (rhesus monkeys) and 0.4 ml emulsion (common marmosets) into the dorsal skin, distributed over 10 spots (rhesus monkeys) and four spots (common marmosets). Clinical signs of arthritis were recorded daily by monitoring of behavioral changes or pain. Twice per week the monkeys underwent a complete physical inspection of all the joints for redness and/or swelling using the integrated discomforted scoring scheme [13]. Late-stage sera were collected from all monkeys and used for CII-specific analysis.

### Induction and evaluation of CIA in mice

Rat CII was prepared from the SWARM chondrosarcoma by pepsin digestion as described previously [14,15]. For induction of chronic arthritis, B10.Q × (BALB/c × B10.Q) N2 mice (*n* = 10) were immunized with 100 μg rat CII in 0.1 M acetic acid, emulsified in incomplete Freund’s adjuvant (Difco). For induction of acute arthritis, B10.Q mice (*n* = 10) were immunized similarly except for the use of complete Freund’s adjuvant (Difco). Both strains were boosted at day 35 with 50 μg rat CII in incomplete Freund’s adjuvant. Clinical scoring of animals was done blindly up to 80 days for acute arthritis and up to 210 days for chronic arthritis using a macroscopic scoring system: 1 point was given for each swollen or red toe or joint and 5 points for a swollen ankle, adding up to a maximum score of 60 points per mouse.

### Antibody measurements

#### Rheumatoid arthritis patients

The synthesis of human triple-helical CII peptides has been described in detail elsewhere [16]. For antibody measurements, enzyme-linked immunosorbent assay plates (NUNC, Roskilde, Denmark) were coated with human CII peptides C1, U1, and J1 (5 μg/ml) at 4°C overnight. After blocking the plates, paired serum and SF samples (diluted 1:100 in radioimmunoassay buffer +10% fetal calf serum) were added followed by incubation for 2 hours at room temperature. Horseradish peroxidase-conjugated goat anti-human IgG (Jackson Immuno Research Laboratories, West Grove, PA, USA) was then added, and interaction with bound antibodies was detected using the chromogenic substrate TMB (Sigma-Aldrich St. Louis, MO, USA). Plates were read at 450 nm with a reference of 650 nm. A standard curve from a positive serum to specific antigen was included on each plate and was further used to translate optical density values to arbitrary units. The cutoff was determined as the mean ± three standard deviations using the control cohort of 100 healthy individuals. To be able to include all analyzed individuals in the graphs, negative responders were given a value of 0.1, which is below the detection limit for positivity. The total IgG concentration (g/l) was determined at the Clinical Immunology Laboratory, Karolinska Hospital, Solna, using commercial radial immune-diffusion assays (Dade-Behring, Marburg, Germany) and rate nephelometry (IMMAGE Immunochemistry System; Beckman Coulter, Fullerton, CA, USA). The assays were calibrated against the international standard CRM470. The paired SF samples were normalized based on the values for total IgG.

#### Nonhuman primates

Enzyme-linked immunosorbent assay plates (NUNC) were coated with rat CII (10 μg/ml) and the synthetic CII peptides C1, U1, and J1 (5 μg/ml) at 4°C overnight. After blocking the plates, serum samples (diluted 1:400) from arthritic CIA monkeys were added and incubated for 2 hours in room temperature. Alkaline phosphatase-conjugated goat (fab)_2_ anti-monkey IgG (Invitrogen, Carlsbad, CA, USA) was then added to the bound antibodies, which were detected with polynitrophenylphosphate diluted in Tris buffer (Sigma Aldrich, St. Loius, MO, USA). Absorbance at 405 nm was measured with a Synergy 2 plate reader (BioTek Instruments). The cutoff value for the assay was calculated as the mean absorbance value of the antibody response to the CII epitopes from naive monkeys ± three standard deviations (calculated to 0.11). All absorbance values below this cutoff value were considered negative. The serum dilution required for each primate to reach the cutoff absorbance value was calculated. All animals with antibody titers below the detection limit were assigned a value of 1.

#### Statistical analysis

One-way analysis of variance (Kruskal–Wallis test) followed by Dunn’s multiple comparison tests were used to analyze the differences between RA and healthy control groups. A paired *t* test (Wilcoxon signed-rank test) was used for comparison between serum and SF antibody levels. *P* < 0.05 was considered statistically significant. Quantitative data are expressed as the mean ± standard error of the mean, and significance analysis was calculated using the Mann–Whitney test.

## Results

### A collagen peptide library generated for systematic epitope mapping of collagen type II

**Figure 1:**
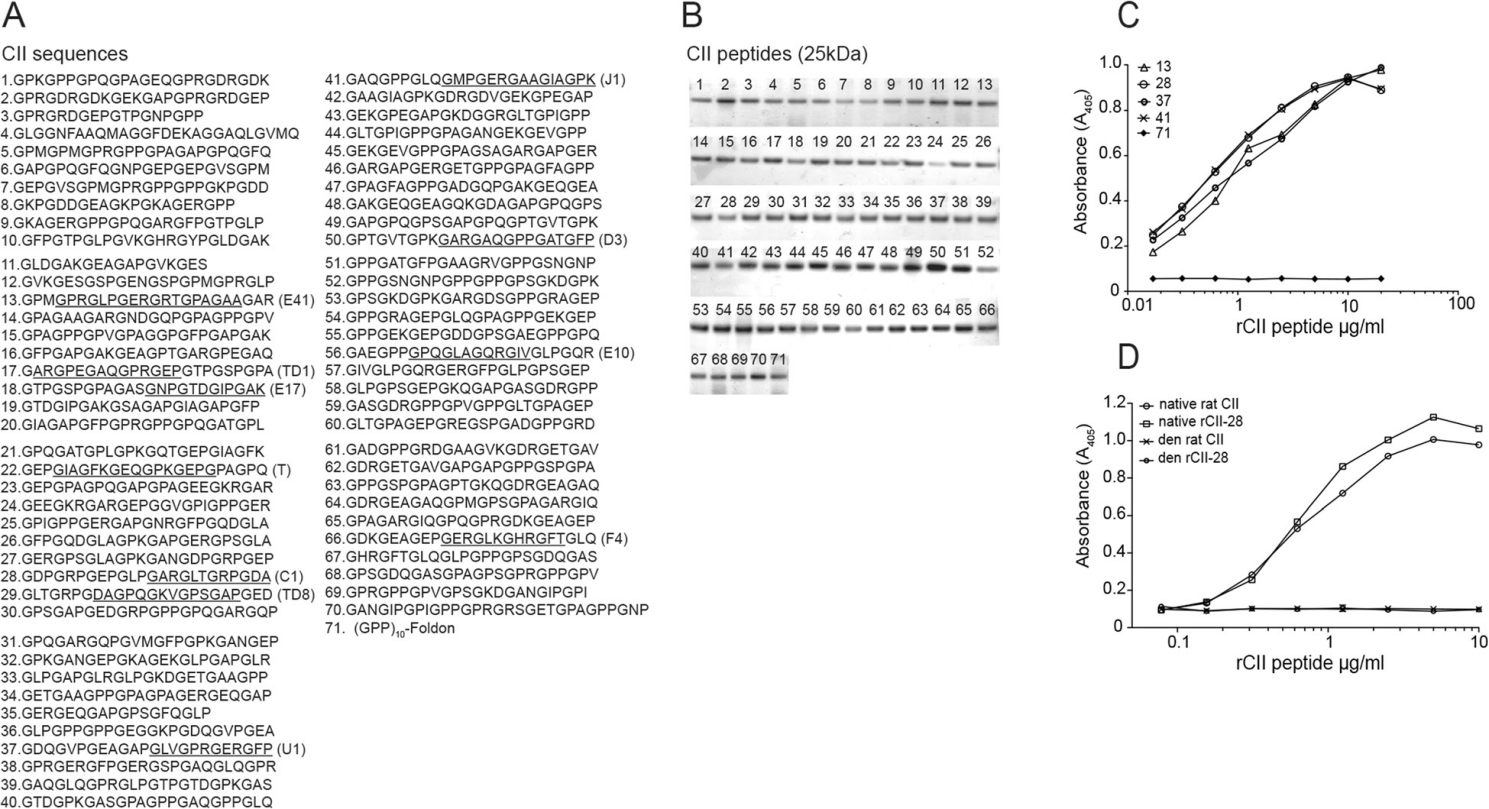
**Generation of a recombinant collagen type II peptide library. (A)** Amino acid sequences of the inserted collagen type II (CII) sequences into the GPP-Foldon scaffold and the corresponding peptide numbers (1 to 71). Underlined letters correspond to known B-cell and T-cell epitopes followed with their names in brackets. The control peptide (rCII-71) lacks the CII insert but includes the Foldon trimerization domain and GPP_10_. **(B)** Sodium dodecyl sulfate polyacrylamide gel electrophoresis showing protein bands at 25 kDa for all 71 rCII peptides. **(C)** Enzyme-linked immunosorbent assay confirming monoclonal antibody recognition to four of the immunodominant rCII peptides (122:41 specific for rCII-13, CIIC1 specific for rCII-28, the UL1 antibody specific for rCII-37, M2139 recognizing rCII-41) and testing of the monoclonal antibody mix to the control peptide rCII-71. **(D)** Monoclonal antibody CIIC1 binds to triple-helical but not heat-denatured rCII-28 peptides and rat CII. rCII, recombinant collagen type II.

The human CII expression constructs used in this analysis were based on the work of Engel and coworkers [17,18] and the preparation and purification is further explained in Additional file 1. In total, 70 recombinant human CII (rCII) constructs were generated covering the entire triple-helical domain of CII and the N-terminal collagen-like sequence of human CII (Figure 1A). There are several epitopes that have been discovered in CIA in mice and these are marked in Figure 1A. C1, U1, and J1 are the most studied epitopes, and antibodies specific for these epitopes induce arthritis in mice [5-8,19,20]. Following expression and purification, the rCII peptides were analyzed by sodium dodecyl sulfate polyacrylamide gel electrophoresis, where each peptide band corresponded to the calculated mass of the triple-helical CII peptides (~25 kDa) (Figure 1B). We confirmed that our monoclonal antibodies were conformation dependent; that is, they were restricted to binding the triple-helical CII epitopes in the triple-helical state only, and bound to the rCII peptides containing their specific epitope (Figure 1C). We could also show that antibody binding to the rCII peptides was completely abolished upon heat denaturation of the peptides (Figure 1D).

### Identification of several new collagen type II epitopes and epitope spreading in arthritic mice

**Figure 2:**
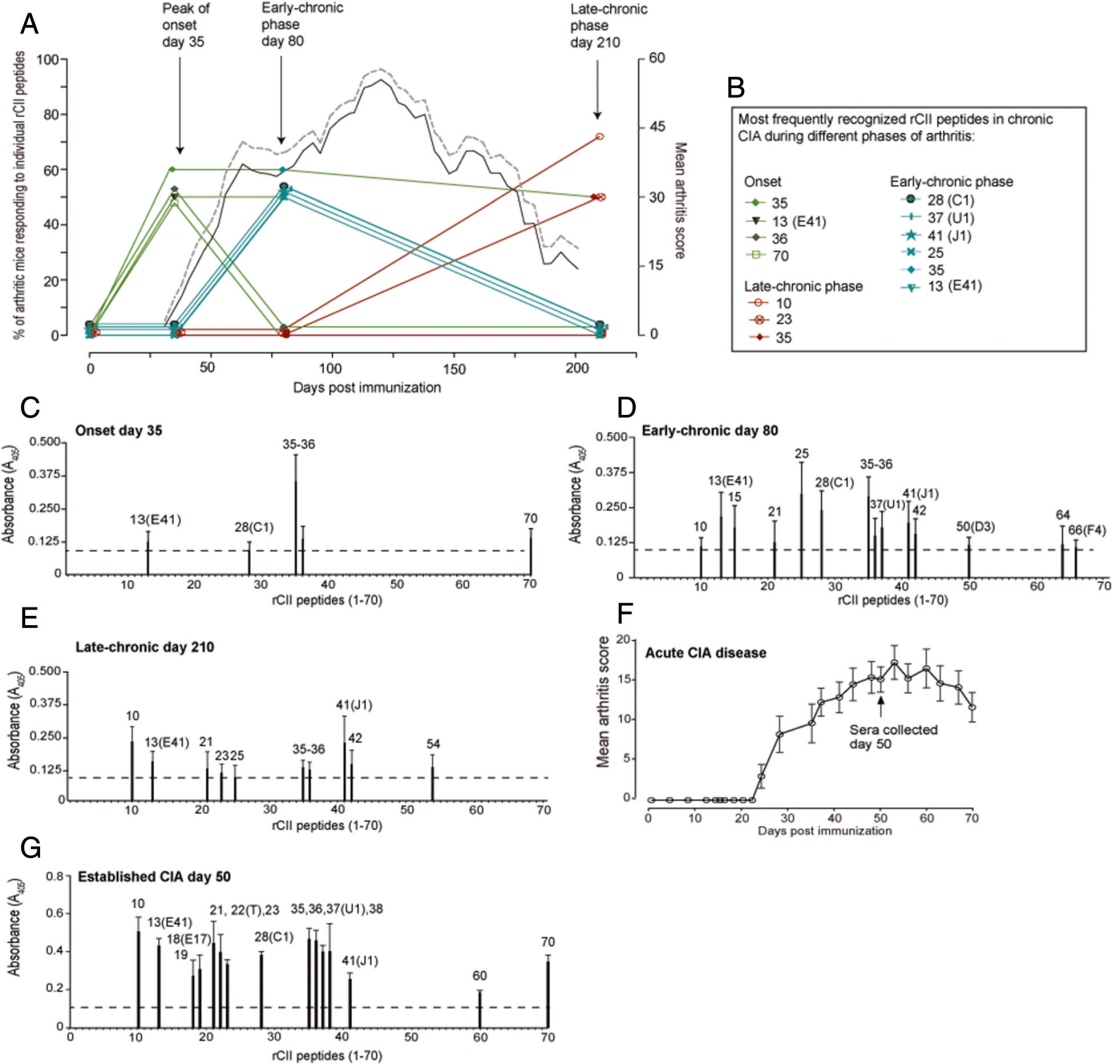
**Specific antibody responses to recombinant collagen type II peptides in chronic and acute collagen-induced arthritis. (A)** Chronic arthritis in male mice from B10.Q × (BALB/c × B10.Q) N2 mice (*n* = 10). Clinical arthritis is denoted on the right *y* axis: straight lines, mean arthritis score; dashed grey lines, mean + standard error the mean. Scoring of the mice was performed until day 201. The left *y* axis shows percent responders to the overlapping recombinant collagen type (rCII) peptides in sera from chronic collagen-induced arthritis (CIA) mice at day 35 (onset), day 80 (early-chronic disease), and day 210 (late-chronic disease). **(B)** rCII peptides detected in ≥50% of the mice are included. **(C)**, **(D)**, **(E)** Autoantibody recognition of rCII peptides in chronic CIA: dotted lines, cutoff calculated as the mean ± three standard deviations of naïve mice. Numbers above the bars correspond to the rCII peptides in the library followed by the epitope name in brackets for B-cell epitopes identified earlier. **(F)** CIA development in B10.Q male mice depicted as the mean arthritis score + standard error of the mean. **(G)** Sera from the acute CIA mice, collected on day 50, were also tested for specificity to the rCII peptides.

To follow the CII-specific antibody response throughout different disease phases we used a mouse strain in which CIA persists chronically, with a relapsing joint inflammation, for over 200 days (Figure 2A) [21]. Arthritis development was monitored and sera were collected at three time points (days 35, 80 and 210). The antibody reactivity of the collected sera to the generated rCII peptide library identified 10 rCII peptides that were frequently recognized, and antibody specificity differed between the different disease phases (Figure 2A,B). Few rCII peptides were recognized at disease onset (Figure 2C), whereas the number of recognized epitopes dramatically increased around early-chronic arthritis (Figure 2D) and slightly decreased in the late-chronic phase (Figure 2E). The rCII peptides recognized included, among others, the disease-related rCII-28, rCII-37, and rCII041, which harbor the major CII epitopes C1, U1, and J1. To investigate the epitope-specific response to that of another arthritis model we followed acute CIA in B10.Q male mice and collected sera at day 50 for CII-specific analysis (Figure 2F). Also here, during the established disease (Figure 2G) the major CII epitopes C1, U1, and J1 were prominent together with several other both earlier identified and new CII epitopes.

### Antibody responses to C1, U1, and J1 are prominent in two monkey species with CIA

**Figure 3:**
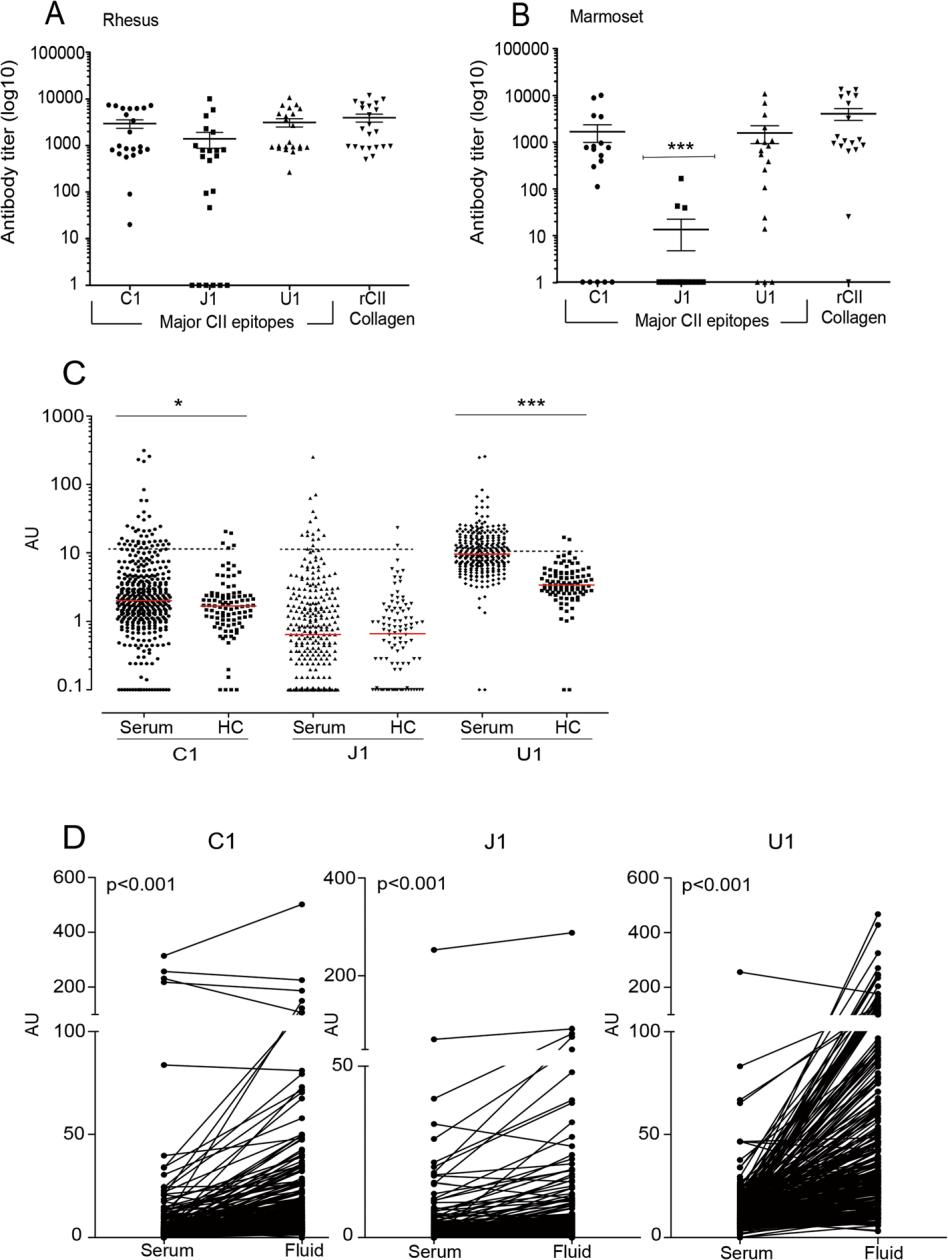
**Epitope-specific response in human rheumatoid arthritis and nonhuman primate species. (A)**, **(B)** Antibody responses to the major collagen type II (CII) epitopes and to entire triple-helical rat CII in nonhuman primates (rhesus monkeys and common marmosets). The serum dilution required for each individual to reach the cutoff absorbance value was calculated and is depicted on the *y* axis. All animals with antibody titers below the detection limit were assigned a value of 1. **(C)** Antibody responses to major CII epitopes in sera from rheumatoid arthritis (RA) patients and healthy control (HC) subjects. All individuals with antibody titers below the detection limit for positivity were assigned a value of 0.1. Each data point represents one individual: dotted lines, cutoff value for positivity; horizontal red lines, median. **(D)** Antibody responses to major CII epitopes in sera and synovial fluid (SF) from RA patients. After normalization based on the values for total IgG, the levels of anti-C1, anti-U1, and anti-J1 antibodies were determined in paired samples of serum and SF from 290 RA patients. AU, arbitrary units. *P < 0.05, ***P < 0.001. Values are the mean ± standard error of the mean.

To make comparative analyses in nonhuman primates we selected two species, the rhesus macaque (*M. mulatta*) and the common marmoset (*C. jacchus*). These developed CIA after immunization with chicken-derived CII [13,22]. The arthritic nonhuman primates had high serum antibody responses towards rat CII, and sera from all 22 rhesus monkeys responded strongly to the major epitopes C1 and U1 (Figure 3A). Sera from the majority of rhesus monkeys (16 out of 22 samples) showed a high response also towards epitope J1. In contrast, sera from the common marmosets displayed a broader variation in responses against the two major epitopes C1 and U1, with more negative samples (Figure 3B). Positive responses to J1 were only detected in three out of the 18 common marmosets.

### High frequencies of antibodies to major collagen type II epitopes were observed in RA serum and synovial fluid samples

For analyzing the response in RA we used a previously described cohort of established RA with both sera and SF from the same patients [11], using an approach where small CII triple-helical peptides of C1, U1, and J1 were synthesized according to a protocol described by Grab and colleagues [23]. We found that the antibody levels against all three CII epitopes were higher in RA patients compared with healthy controls, and the SF patient samples had higher titers than the paired serum samples (Figure 3C,D). In addition, the frequency of patients positive for the C1 and U1 epitopes in SF also increased in comparison with sera: 23% versus 13% and 77% versus 47% for C1 and U1, respectively.

### Anti-collagen type II antibody responses differ from the anti-citrullinated protein antibody response

To investigate whether the anti-CII antibodies define a different subset of RA we used earlier data on the same cohort [11] to correlate with the ACPA response. As summarized in Table 1, all anti-CII antibodies show low *r* values for correlation with cyclic citrullinated peptide (CCP), either in serum or SF. In comparison, antibodies against the triple-helical C1 peptide citrullinated at both arginine positions (that is, CitC1) positively correlate with CCP in serum and SF (*r* = 0.655 and *r* = 0.643 respectively). Furthermore, in contrast to ACPA that are confined to the CCP-positive RA subset, anti-CII antibodies were found in both the CCP-positive and CCP-negative subsets. While only 6% of CCP-negative patients have anti-CitC1 antibodies in sera, 13% have antibodies towards C1 and 38% towards U1. In SF 5% of the CCP-negative patients have antibodies against CitC1, whereas 13% and 63% have antibodies against C1 and U1, respectively. The distribution of anti-CII antibodies in these two subsets is further illustrated in Figure S1A,B in Additional file 2.

**Table 1:** Correlation and frequencies between collagen type II epitopes and CCP

**Collagen type II epitope**	**Correlation with CCP ( *r *)**		**Frequency in sera (%)**		**Frequency in synovial fluid (%)**	
	**Serum**	**Fluid**	**CCP**^**–**^	**CCP**^**+**^	**CCP**^**–**^	**CCP**^**+**^
C1	0.076	0.211	12.5	12.9	13.2	27.1
J1	0.012	0.130	8.8	5.7	7.7	5.5
U1	0.153	0.395	37.5	50.5	62.6	82.9
CitC1	0.655	0.643	6.2	53.8	5.5	49.7

CCP, cyclic citrullinated peptide.

We further examined whether CII antibodies follow ACPA association with HLA-SE, in particular with HLA-DRB1*04. We therefore divided our patients into three different groups based on the identity of their SE alleles: those who do not carry the SE alleles (*n* = 47, -/–), patients carrying one or two copies of DRB1*01 alleles (*n* = 50, DR*01), and patients carrying one or two copies of DRB1*04 alleles (*n* = 127, DR*04). To better stratify the analysis, patients carrying both the DRB1*01 allele and the DRB1*04 allele (*n* = 40) were excluded from this analysis. In contrast to ACPA levels that strongly associate with HLA-DRB1*04, the CII examined antibody level association was less pronounced with HLA-DRB1*04 or other SE alleles (Table 2; Figure S2A,B,C,D,E,F in Additional file 3).

**Table 2:** Odds ratio for the association of antibody-positive patients within the different HLA-DRB1 groups

	**No SE (*n*** **= 47)**	HLA-DRB1*01 (*n* **= 50)**		HLA-DRB1*04 (*n* **= 127)**		
**Antigen**	**Frequency of positive patients (%)**	**Frequency of positive patients (%)**	Odds ratio DR*01 vs. no SE	**Frequency of positive patients (%)**	Odds ratio DR*04 vs. no SE	Odds ratio DR*04 vs. DR*01
C1 sera	14.9	16	1.088, 95% CI 0.3613 to 0.280	11.8	0.7653, 95% CI0.291 to 2.013	0.703, 95% CI 0.278 to 1.780
C1 SF	10.6	22	2.369, 95% CI 0.755 to 7.436	25.2	2.829, 95% CI 1.030 to 7.771	1.194, 95% CI 0.547 to 2.605
J1 sera	6.4	14	2.388, 95% CI 0.579 to 9.845	5.5	0.856, 95% CI 0.212 to 3.457	0.3583, 95% CI 0.119 to 1.081
J1 SF	6.4	8	1.275, 95% CI 0.361 to 3.280	7.1	1.119, 95% CI 0.289 to 4.324	0.469, 95% CI 0.164 to 1.336
U1 sera	42.6	38	0.827, 95% CI 0.367 to 1.865	48	1.227, 95% CI 0.624 to 2.413	1.483, 95% CI 0.759 to 2.898
U1 SF	68.1	82	2.135, 95% CI 0.828 to 5.506	76.4	1.516, 95% CI 0.7245 to 3.169	0.710, 95% CI 0.310 to 1.627
CitC1 sera	27.6	26	0.919, 95% CI 0.374 to 2.258	53	2.921, 95% CI 1.410 to 6.049	3.178, 95% CI 1.544 to 6.541
CitC1 SF	17	26	1.713, 95% CI 0.637 to 4.606	48.8	4.650, 95% CI 2.014 to 10.74	2.715, 95% CI 1.319 to 5.586

CI, confidence interval; SF, synovial fluid.

## Discussion

In this study we demonstrate that anti-CII antibodies, in particular antibodies directed to the major epitope U1, are frequent and present at the site of inflammation in RA. A significant higher antibody response against the U1 epitope was observed in SF (77%) compared with levels in sera (47%), supporting the notion of an increased immune response to locally released CII in the joints. This suggests that anti-CII antibodies appear around the clinical onset and are likely to play a role in the disease process. Antibodies to the investigated three CII epitopes are all shown to be highly arthritogenic in mouse models of RA and one of the first steps observed in the joint pathology is antibody-induced destabilization of cartilage [8]. This observation could open up this tissue for a subsequent inflammatory attack.

We observed a higher prevalence of CII-specific antibodies directed against U1 using our triple-helical peptides compared with previous studies using CII as antigen [2,4]. There are several possible explanations for the higher frequency of CII-positive patients when using defined triple-helical peptides as compared with CII isolated from cartilage. Firstly, semi-purified CII derived from tissue is in complex with other matrix proteins, and also with pepsin used for tissue extraction. These contaminants could very probably block potential CII epitopes. Secondly, crosslinking with fibrinogen or denaturation of CII *in vitro* is likely to disturb assays using semi-purified CII. Thirdly, the smaller triple-helical CII epitopes are coated on enzyme-linked immunosorbent assay plates at higher molar densities than the large CII protein, resulting in capture of antibodies with lower affinity.

Using our assay with defined triple-helical CII epitopes possibly allowed detection of antibodies with lower affinity, and this may have a large effect on the detection rate. Importantly, the anti-CII antibody response in mice, even antibodies that are pathogenic, are to a large extent germline encoded and thereby lack somatic mutated, generated affinity maturation [6,7,24,25]. The observed anti-CII antibody response was not confined to the CCP-positive subset of RA and did not significantly associate with specific HLA-DR alleles. The lack of HLA-DR association could, however, be due to low statistical power and because we used a cohort with more established RA, since earlier investigations into early RA have suggested a weak association with DRB1*0401 [26,27].

Despite the genetic differences between rodents, monkeys, and humans and the fact that the experimental arthritis was induced through immunization with CII in the various species leading to strong anti-CII antibody responses, the fine specificity of the major epitopes was still largely conserved. The only exception was the lack of response to the J1 epitope in the common marmoset. This lack is in fact similar to the mouse in which the response to J1 is strain specific [28]. Interestingly, the heterogeneity in the mouse has been mapped to the variable heavy chain locus, indicating that constraints of the binding structures lead to restrictions in using allele-specific V genes [7]. The analysis of the triple-helical peptide CII library showed that numerous epitopes could be targeted by autoantibodies and responses against different CII epitopes vary at different stages of the disease. The generated CII peptide library revealed that the three more thoroughly investigated major CII epitopes are present in both acute and chronic stages of arthritis. The binding site of a CII-binding antibody is often the site for binding of matrix proteins (reviewed in [10]), which could explain why the epitope specificity of the antibodies is important for their pathogenicity. CII provides numerous epitopes that are recognized by antibodies in RA but there are also other cartilage protein epitopes to be detected. This leads to the possibility that cartilage is frequently recognized in RA and that these antibodies could play an important role in the disease. Assays to detect such antibodies would therefore provide valuable information, in particular to predict and follow the joint inflammatory attack. Further dissection of the CII epitopes, and their citrullinated counterparts [29,30], could be of importance to understand the pathogenic and regulatory pathways in RA.

## Conclusions

Antibody responses against triple-helical CII epitopes are common in RA, and the high antibody titers in SF indicate that these are likely to originate from rheumatoid joints. However, there was no correlation with the ACPA response. Importantly, the fine specificity of the anti-CII response is largely conserved between RA in humans and CIA in monkeys and rodents where the recognized epitopes have a major pathogenic role. In conclusion, our results suggest that anti-CII antibodies are likely to reflect a pathway other than ACPA and may both contribute to, as well as be the consequence of, local joint inflammation.

## Abbreviations

ACPA: anti-citrullinated protein antibodies; CCP: cyclic citrullinated peptide; CIA: collagen-induced arthritis; CII: collagen type II; RA: rheumatoid arthritis; rCII: recombinant human collagen type II; SF: synovial fluid.

## Competing interest

RH is co-inventor of a patent protecting the use of collagen triple-helical epitopes for diagnostic use. The remaining authors declare that they have no competing interests.

## Authors’ contributions

RH made the conceptual outline of the study. IL, EL and KSN collected and analyzed the rodent data. OS collected and VM analyzed the human data. MV and Bt’H collected and IL analyzed the primate data. HU made the recombinant collagen proteins, and HU together with IA helped with running assays and the analysis of data. IL wrote the first draft of the manuscript. All authors read, improved and approved the final manuscript. RH coordinated and supervised the overall study.

## Supplementary Material

Additional file 1 is a description of supplementary materials and methods for CII peptide design, expression and purification of recombinant CII peptides, and antibody measurements in mice sera with recombinant CII peptides, rhesus monkeys and common marmosets. Click here for file

Additional file 2 is Figure S1A and S1B showing the distribution of anti-CII antibodies in sera and in synovial fluid of CCP-positive and CCP-negative RA subsets. Click here for file

Additional file 3 is Figure S2A,B,C,D,E,F showing the association between CII antibodies and HLA-SE alleles, in particular HLA-DRB1*04. The patients were divided into three different groups based on the identity of their SE alleles: those who do not carry the SE alleles (*n* = 47, -/-), patients carrying one or two copies of DRB1*01 alleles (*n* = 50, DR*01), and patients carrying one or two copies of DRB1*04 alleles (*n* = 127,DR*04). To better stratify the analysis, patients carrying both the DRB1*01 allele and the DRB1*04 allele (*n* = 40) were excluded from this analysis. AU, arbitrary units. Click here for file
