# Supplementary material for: Type II collagen antibody response is enriched in the synovial fluid of rheumatoid joints and directed to the same major epitopes as in collagen induced arthritis in primates and mice
Source: Arthritis Res Ther. 2014 Jul 8;16(4):R143. doi: 10.1186/ar4605 (PMC4226996; doi:10.1186/ar4605)
Supplement: Additional file 1 — is a description of supplementary materials and methods for CII peptide design, expression and purification of recombinant CII peptides, and antibody measurements in mice sera with recombinant CII peptides, rhesus monkeys and common marmosets. [file ar4605-S1.docx]

**Supplementary material and methods**

**CII peptide design**

The sequence of the triple-helical segment of human CII was divided into 70 overlapping fragments, with the majority of them being 24 amino acids long. The N-terminal collagen like sequence covers fragments 1-3, while fragment 4 contains the proteolytic cleavage site. The mature CII sequence starts with fragment 5. With a few exceptions, each fragment overlaps by nine amino acid residues with the previous and the next peptide in the series. To achieve triple-helical conformation of the encoded peptides, five consecutive GPP triplets ((GPP)_5_) were added to both N- and C-terminus of each CII sequence, followed by a fusion of the foldon trimerization domain (Frank et al. 2001; Stetefeld et al. 2003) to the C-terminus. The expression constructs were synthesized and subcloned into pET-28 (Novagen) expression vector by a commercial gene synthesis service (Eurofinns MWG Operon, Germany). In summary, each CII sequence fragment was expressed with surrounding (GPP)_5_ sequences, an N-terminal His_6_-tag followed by a thrombin cleavage site (MGSSHHHHHHSSGLVPRGSHMG), and a foldon trimerization domain (SGYIPEAPRDGQAYVRKDGEWVLLSTFL) at the C-terminus.

**Expression and purification of recombinant CII peptides**

The constructs were transformed into chemically competent BL-21 (DE3) (Novagen) bacterial cell line. All 70 bacterial clones and a control were grown in a 96-well deep well plate (Whatman) containing 1 ml of the Luria Broth (LB) supplemented with kanamycin (50 μg/ml). The plate was sealed with an air-porous plate cover (ThermoScientific) and incubated at 37ºC under agitation (225 rpm) overnight. 100 μl of the overnight cultures were transferred to 5 ml of the Terrific Broth (TB) containing kanamycin (50 μg/ml) in 24-well deep well plates (Whatman), and grown at 37°C, 225 rpm until the OD_600_ reached at least 0.6. Expression was induced with 1mM isopropyl β-D-1-thiogalactopyranoside (IPTG). Concomitantly, the temperature was decreased to 22°C. After an overnight growth, the cells were centrifuged at 3000 g for 20 min at 4°C, and the bacterial cell pellets were re-suspended in 300 μl Bugbuster (Novagen) solution containing protease inhibitor tablets (Roche) and benzonase (Novagen). The lysates were stored at -80°C. Prior to purification, the samples were thawed and centrifuged at 3500 g for 20 min. The supernatants subsequently were transferred to a multi-screen 0.65 μm filter plate (Millipore) placed on top of a sterile collection plate (300 μl/well) and centrifuged at 2000 g for 10 min. For metal-ion affinity based purification of the his-tagged recombinant CII peptides (rCII) the supernatants were transferred to a new 96-well collection plate containing 50 μl Dynabeads (Invitrogen) placed on a DynaMag^TM^ plate. The purification was accomplished according to manufacturers instruction. For buffer exchange into phosphate buffered saline (PBS), the eluted rCII peptides were transferred to a 96-well microdialysis plate (Pierce ThermoScientific). The concentrations of the rCII were calculated using the BCA protein assay (ThermoScientific). The purified rCII peptides were analyzed in 4-12 % bis-tris gels (Novex, Invitrogen). Peptides rCII-28 and rCII-71 were subsequently transferred to a polyvinylidene fluoride (PVDF) membrane. Immunodetection of the peptides was achieved using a pool of polyclonal arthritic serum and autoradiography film processing equipment as described earlier (Dobritzsch et al. 2011).

Purified rCII peptides were coated on ELISA plates (NUNC, Roskilde, Denmark). After blocking of the plates with 2% (w/v) milk powder (Semper, Sundbyberg, Sweden), four anti-CII mAbs of well-defined specificity were tested for their capacity to bind the rCII peptide library: 122:41 (Wernhoff et al. 2001), CIIC1 (Holmdahl et al. 1986), UL-1 (Bajtner et al. 2005) and M2139 (Mo and Holmdahl 1996).

**Antibody measurements in mice sera using recombinant CII peptides**

Diluted serum samples (1:400), collected from the two mice strains B10.Q (day 50, n=10) and B10.Q x (Balb/c x B10.Q) N2 (day 35, 50, 200, n=10) after CIA induction, were added to ELISA plates (NUNC, Roskilde, Denmark) pre-coated with the 71 rCII peptides. Specific binding was detected using an HRP-conjugated anti-mouse IgG H+L chain-specific secondary antibody (Jackson Immuno Research Laboratories, West Baltimore, USA) and the chromogenic peroxidase-substrate ABTS (Boehringer, Mannheim, Germany). Absorbance at 405 nm was measured with a Synergy 2 plate reader (BioTek Instruments). The mean±SEM absorbance values were calculated using GraphPad Prism version 5 software. The cutoff value was defined by calculating the mean value of the antibody response obtained from naïve mice to the rCII peptides ± 3 standard deviations (SD).
