# Supplementary figures and images for: Type II collagen antibody response is enriched in the synovial fluid of rheumatoid joints and directed to the same major epitopes as in collagen induced arthritis in primates and mice
Source: Arthritis Res Ther. 2014 Jul 8;16(4):R143. doi: 10.1186/ar4605 (PMC4226996; doi:10.1186/ar4605)

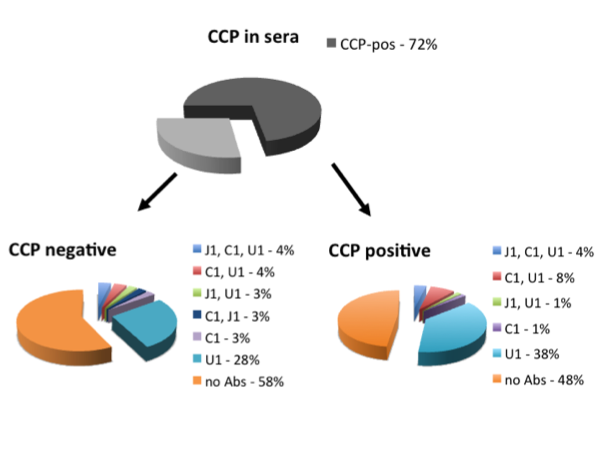

Supplement: Additional file 2 — is Figure S1A and S1B showing the distribution of anti-CII antibodies in sera and in synovial fluid of CCP-positive and CCP-negative RA subsets. [file ar4605-S2.zip › 7487113341205563_add2.png]

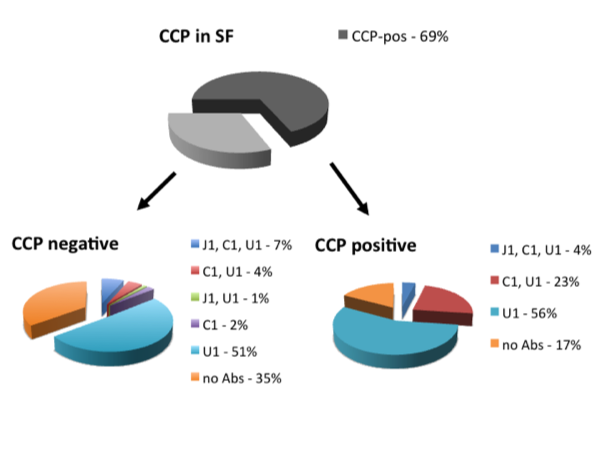

Supplement: Additional file 2 — is Figure S1A and S1B showing the distribution of anti-CII antibodies in sera and in synovial fluid of CCP-positive and CCP-negative RA subsets. [file ar4605-S2.zip › 7487113341205563_add3.png]

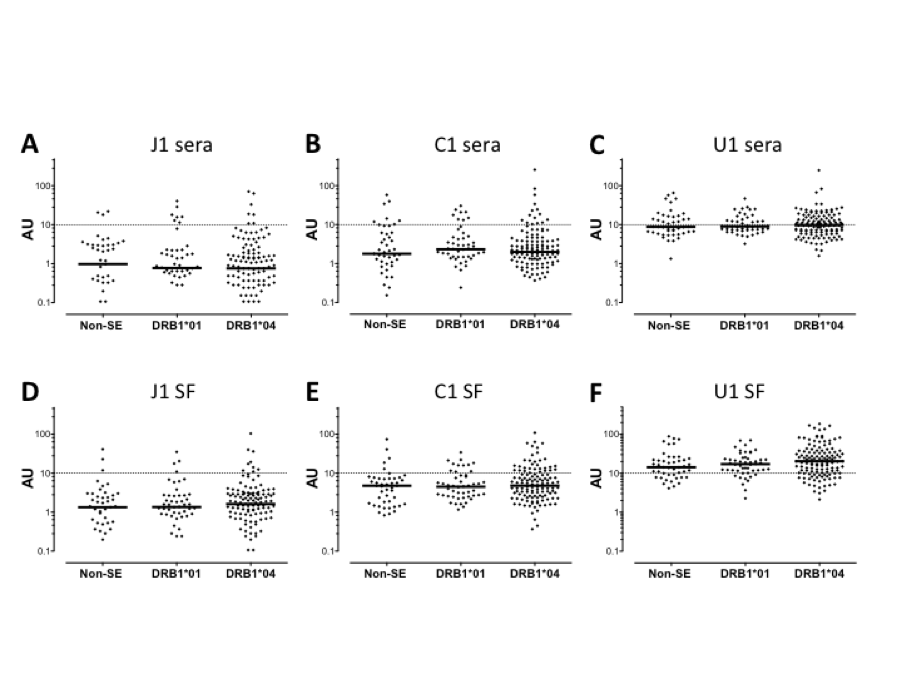

Supplement: Additional file 3 — is Figure S2A,B,C,D,E,F showing the association between CII antibodies and HLA-SE alleles, in particular HLA-DRB1*04. The patients were divided into three different groups based on the identity of their SE alleles: those who do not carry the SE alleles (n = 47, -/-), patients carrying one or two copies of DRB1*01 alleles (n = 50, DR*01), and patients carrying one or two copies of DRB1*04 alleles (n = 127,DR*04). To better stratify the analysis, patients carrying both the DRB1*01 allele and the DRB1*04 allele (n = 40) were excluded from this analysis. AU, arbitrary units. [file ar4605-S3.png]
